# Long-Term Follow-Up of Omental Cranial Transposition to Bypass the Blood-Brain Barrier for Recurrent Glioblastoma: A Case Report and Scientific Rationale

**DOI:** 10.7759/cureus.81566

**Published:** 2025-04-01

**Authors:** Souvik Singha, Julianna Cavallaro, Tamika Wong, Olivia Albers, Samer Ali, Avraham Zlochower, Robert Andrews, John Boockvar

**Affiliations:** 1 Department of Neurosurgery, Lenox Hill Hospital, Donald and Barbara Zucker School of Medicine at Hofstra/Northwell, New York, USA; 2 Department of Pathology, Lenox Hill Hospital, Donald and Barbara Zucker School of Medicine at Hofstra/Northwell, New York, USA; 3 Department of Radiology, Lenox Hill Hospital, Donald and Barbara Zucker School of Medicine at Hofstra/Northwell, New York, USA; 4 Department of General Surgery, Lenox Hill Hospital, Donald and Barbara Zucker School of Medicine at Hofstra/Northwell, New York, USA

**Keywords:** blood brain barrier, clinical trial, glioblastoma, omental cranial transposition, omentum, overall survival

## Abstract

Recurrent glioblastoma multiforme (GBM) is associated with a very poor prognosis due to the limited efficacy of existing therapies and constraints of blood-brain barrier (BBB) permeability. Particularly in the recurrent setting, there is no uniform standard of care treatment. In one of the proposed treatments for recurrent GBM, a laparoscopically harvested omental free tissue autograft is used to favor neovascularization and bypass the BBB, in which patients receive an autologous abdominal omental tissue intracranially following attempted gross total resection of the tumor. In this paper, we report the long-term survival and follow-up data of a patient who underwent this procedure. The progression-free and overall survival were 21 months and 30 months, respectively. We hypothesize that neovascularization from the omental flap helps bypass the BBB, and omental-derived autologous immune cells help penetrate the tumor microenvironment and recognize tumor-associated antigens, thus providing better tumor control for recurrent GBM.

## Introduction

Glioblastoma multiforme (GBM) is the most common malignant brain tumor in humans. It has a poor median survival of 12-15 months and progression-free survival of about five to six months [1]. Most GBMs typically recur within 6-9 months, and the median overall survival from first recurrence is only 3-9 months [2]. Currently, there is no uniform standard of care for recurrent GBM. A major challenge of GBM treatment is the impenetrability of the blood-brain barrier (BBB) and its high tumor cellular heterogeneity. The BBB is a semi-permeable yet highly restrictive separation between blood vessels and the brain, which prevents both large and small-molecule drugs from penetrating tumor cells in the brain [3,4]. Additionally, it hinders the immune response to tumor-associated antigens in GBM [5,6]. In addition, surgical resection of GBM proves to be ineffective in fully eradicating the disease because of its infiltrative nature into the surrounding brain tissue. In 2021, our group established early results of a trial termed “Laparoscopically Harvested Omental Free Tissue Autograft to Bypass the BBB in Human Recurrent GBM” in which patients with recurrent GBM receive an autologous abdominal omental tissue autograft placed intracranially following gross total resection of the tumor [1]. Following tumor resection, the omentum is harvested from the abdomen using laparoscopic surgery and translocated into the resection cavity and connected to the blood vasculature to provide circulation. We hypothesized that omental tissue may provide local neovascularization with blood vessels that lack an intact BBB while also changing the tumor microenvironment and allowing autologous immune cells in the omentum (“milky spots”) to recognize tumor-associated antigens in the region [1]. This exposure should theoretically allow better immune surveillance of the central nervous system tumor, which is otherwise immune-privileged [1,7-8]. This is not the first time omentum has been used for cranial transposition in humans. Increased metabolic support from omentum for deteriorating neurons has been used to treat Alzheimer’s disease [1]. Omentum cranial transposition has also been used to treat patients with stroke or moyamoya disease due to the hypothesis that omentum may improve vascular perfusion [1]. Eligibility for our trial required patients to present with recurrent GBM. Here we report the first long-term survival and follow-up data of a patient who underwent omental cranial transposition for recurrent GBM.

## Case presentation

A 51-year-old male diagnosed with O^6^-methylguanine-DNA methyltransferase (MGMT) methylated, isocitrate dehydrogenase (IDH)-wild type central nervous system WHO Grade IV GBM five years ago (initially presented with a 4.7 cm x 3.5 cm left frontal mass), underwent a gross total resection and was treated with radiation therapy and temozolomide (TMZ). Three years later, imaging revealed progression of disease after the patient reported having memory disturbances and processing difficulties with intermediate mild headaches. The patient enrolled in the omental cranial transposition clinical trial for recurrent GBM and underwent a left-sided craniotomy for re-resection of the tumor. Patients with a histologically confirmed recurrence of GBM intraoperatively, in which at least 80% of the tumor can be removed, are eligible for this trial. Additionally, patients must have a Karnofsky performance status of at least 70% before the operation and have had no prior abdominal surgery. The procedure was performed by a team of general surgeons, neurosurgeons, and head and neck surgeons, and the details of the procedure were described by our group in a previous publication [1]. After a gross total resection of the recurrent GBM guided by 5-aminolevulinic acid (5-ALA) fluorescent marker, omentum was laparoscopically harvested from the abdomen and laid into the resection cavity with the gastroepiploic pedicle running down the neck (gastroepiploic vessels were anastomosed with the right superior thyroid artery and a branch of the facial vein), making sure that blood flow was confirmed by Doppler ultrasound [1]. His post-operative care included the placement of a transient right frontal external ventricular drain (EVD) for mild intraventricular hemorrhage and hydrocephalus, mild aphasia, and mild right hemiparesis; the EVD was subsequently removed, and his symptoms improved completely over the next month. He was followed up with serial imaging.

**Figure 1:**
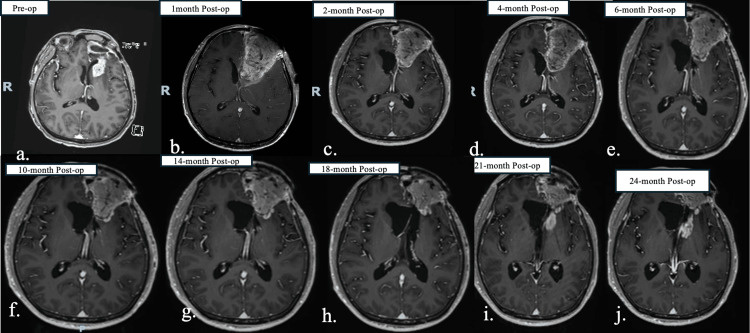
T1 contrast-weighted serial MRI T1 contrast-weighted MRI showing a) pre-operative recurrent left frontal tumor; b) post-operative status with gross total resection of the left frontal tumor with omental cranial transposition without any evidence of disease; c-h) serial follow-up imaging shows stable imaging with progressive shrinkage of the omental flap; i) imaging at the 21st month showing recurrent disease at the left frontal operative site with further shrinkage of the omental flap; j) imaging at the 24th month showing slowly progressive disease.

**Figure 2:**
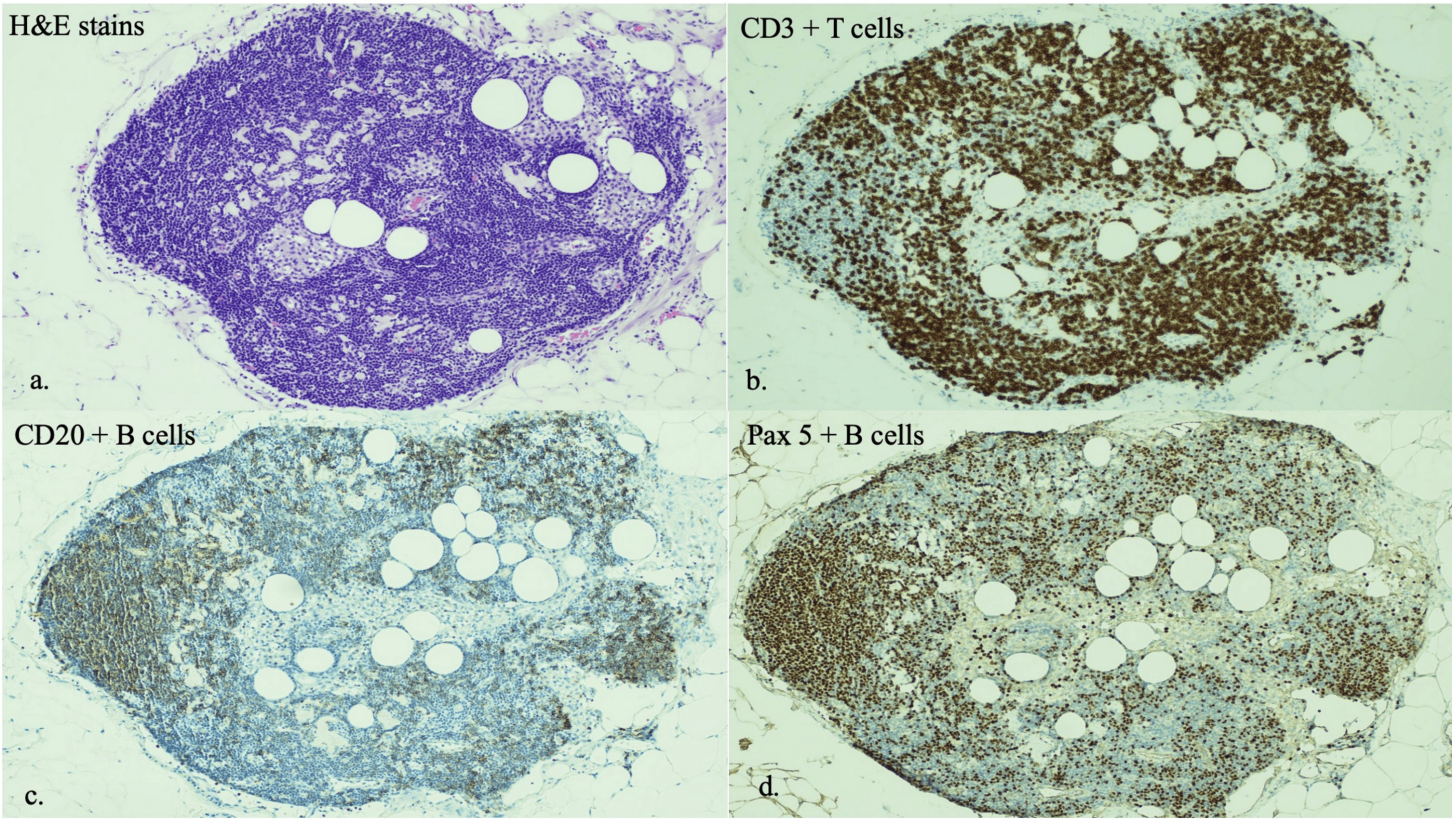
Immunohistochemistry (IHC) staining showing B and T cells a) Hematoxylin and eosin staining of the patient’s omental-derived lymph nodes; b) IHC stains highlighting CD3+ T-cells; c) IHC stains highlighting CD20+ B-cells; d) IHC stains highlighting Pax5+ B-cells.

After intra-cavitary omental transposition, the patient received seven cycles of TMZ and remained disease-free for 21 months. Slowly over time, the omental graft receded in size, likely due to inherent phagocytosis. The MRI at the 21st month showed significant shrinkage of the omental flap and a local small recurrence subjacent to the retracted omental graft (Figure 1). The patient’s Karnofsky performance status was 100, and Eastern Cooperative Oncology Group Performance Status (ECOG PS) was 0 until the 24th-month follow-up visit. He continued as an avid rock climber for two years after surgery. However, his disease slowly progressed, and he refused any further intervention. His general condition started to deteriorate gradually as he became wheelchair-bound and progressively more aphasic. He passed away after 30 months following his omental cranial surgery for recurrent GBM. Post-operative hematoxylin and eosin staining of the patient’s omental-derived lymph nodes showed extensive amounts of CD3+ T-cells, CD20+ B-cells, and Pax5+ B-cells (Figure 2). Radiographically, there was no evidence of the tumor hijacking the omental vascular supply to grow more quickly.

## Discussion

Broadly, the immunosuppressive conditions in GBM result from various factors. These include the immune privilege of the central nervous system due to the presence of the BBB, the concealment of GBM-associated antigens, the limited ability of numerous tumor-killing cells to access the tumor site, and notably, the inhibitory elements affecting tumor-killing cells from diverse sources within the GBM microenvironment [7-9]. This immunosuppressive microenvironment poses a significant challenge in the development of successful immunotherapy for GBM. Despite progress in T cell-based immunotherapy for various tumors, the highly immunosuppressive GBM environment hinders treatment success by impeding T cell function through endogenous inhibition, upregulated checkpoints, immunosuppressive cell infiltration, and impaired T cell migration. Current GBM immunotherapy approaches, including dendritic cell (DC) vaccines, checkpoint inhibitors, and adoptive T-cell therapy (ACT), face challenges due to these inhibitory factors. To address these challenges, several methods have been explored. One promising approach is the transposition of the omental flap, which can achieve our previously stated dual objectives. It can facilitate the ingrowth of multiple blood vessels that lack an intact BBB, and importantly, it can modify the immunosuppressive microenvironment of GBM by recruiting “milky-spot” derived autologous T cells and macrophages. The concept of omental transposition onto the human brain originated in the surgical research laboratory. Initial findings indicated that placing the omentum on the brains of dogs and monkeys resulted in an elevated cerebral blood flow (CBF) originating from neovascularization from the omentum [10]. Subsequent studies demonstrated that blood flow from the omentum was directed into the brain through multiple blood vessels that developed at the omental-cerebral cortex interface. These vessels then extended vertically and deeply into the underlying brain while lacking the ultrastructure of an intact BBB [10]. The fundamental immunologic units within the omentum are termed milky spots. In animals, cells in these milky spots originate from the mononuclear phagocyte system and encompass macrophages (70%), B-lymphocytes (10%), T-lymphocytes (10%), mast cells, and stromal cells (Figure 2). These cellular components are organized around the omental glomeruli situated directly beneath the mesothelium. Supporting these structures is a delicate network of reticular fibers, forming the organ's framework. The omentum houses substantial quantities of B and T lymphocytes, typically found around the periarteriolar vessels [11]. Our patient’s omental lymph nodes showed high levels of CD3+ T-cells, CD20+ B-cells, and Pax5+ B-cells. By transposing omentum into the resected tumor cavity, we hypothesized that these immune cells would get recruited and allow for local tumor immune surveillance of the microenvironment. Additionally, it is hypothesized that the neovascularization in the resection cavity from the omental autograft aids in drug delivery of the patient's post-operative TMZ to achieve local control.

In our patient presented here, we show the world’s first long-term survivor following omental cranial transposition for recurrent GBM. We show that there was no evidence of the tumor hijacking the vascularity provided by the omentum and growing more quickly. To the contrary, tumor recurrence did not occur in the location of the transposed omentum until approximately two years later, when the omentum was partially phagocytosed. We hypothesize that the shrinking of the omentum tissue in the tumor resection cavity was due to macrophage infiltration and omental phagocytosis. Ongoing trials using omental cranial transposition for recurrent GBM will help determine if this treatment is efficacious in prolonging progression-free and overall survival in GBM.

## Conclusions

Recurrent GBM poses one of the most formidable treatment challenges, with a dismal prognosis. Innovative techniques and ideas are imperative to overcome the limitations of traditional treatment options. Our approach attempts to address two critical issues with recurrent GBM: the BBB and the local immunosuppressive tumor microenvironment. The progression-free survival and overall survival rates of 21 and 30 months, respectively, of this patient are indeed promising when compared to historical survival rates. However, further clinical trials with larger numbers will determine if this technique is safe and efficacious for patients with recurrent GBM. The presence of B and T cells in the omental graft suggests potential immune modulation, but further studies are needed to determine the extent of their tumor-fighting activity. Combining omental cranial transposition with various immune checkpoint inhibitors may further unleash omental-derived tumor-infiltrating T cells into the tumor microenvironment.
